# A 3.2–3.6 GHz GaN Doherty Power Amplifier Module Based on a Compact Low-Loss Combiner

**DOI:** 10.3390/mi16020220

**Published:** 2025-02-15

**Authors:** Xiyu Wang, Dehan Wang, Wenming Li, Xiaolin Lv, Kai Cui, Haijun Liu, Kai Kang

**Affiliations:** 1 State Key Laboratory of Mobile Network and Mobile Multimedia Technology, Shenzhen 518055, China; 2 RCH System Design Department, ZTE Corporation, Xi’an 710047, China; 3 School of Electronic Engineering, University of Electronic Science and Technology of China (UESTC), Chengdu 611731, China

**Keywords:** 5G, compact low-loss combiner, Doherty power amplifiers (DPAs), gallium nitride (GaN), module, surface-mounted devices (SMDs), package

## Abstract

In this paper, a 3.2–3.6 GHz two-stage Doherty power amplifier (PA) module is proposed for fifth-generation (5G) massive multiple-input multiple-output (MIMO) base stations. A detailed design method and procedure for a compact and low-loss combiner suitable for the Doherty PA module are introduced. Based on the proposed combiner, a Doherty PA module is implemented using gallium nitride (GaN) transistors and surface-mounted devices (SMDs) with a packaged size of 8 × 8 mm^2^. The proposed two-stage Doherty PA module achieves a 3 dB small-signal bandwidth of 3.1–3.9 GHz and a peak gain of 31.7 dB. From 3.2 to 3.6 GHz, the saturated output power is 40.4–41.1 dBm. Moreover, the measured saturated drain efficiency (DE) and 8 dB power back-off (PBO) DE reach 51–56.6% and 45.5–48.6%, respectively.

## 1. Introduction

With the rapid development of fifth-generation (5G) mobile communications, complex modulation with high peak-to-average power ratios (PAPRs) has been widely used to fulfill the increasing demand for high data rates. Therefore, Doherty power amplifiers (DPAs) are widely used to meet the requirement of exhibiting a high efficiency in the back-off region [1,2]. Meanwhile, the massive multiple-input multiple-output (MIMO) technique has been utilized to further increase the wireless data rate, which requires the size of each PA to be as compact as possible to ensure a reasonable system size [3].

Monolithic microwave integrated circuits (MMICs) can effectively reduce the PA’s size; however, their commercialization is difficult due to their high fabrication costs. On the other hand, the PA module, which is implemented as a hybrid which integrates gallium nitride (GaN) transistors as the power cells while employing low-cost components including gallium arsenide (GaAs) as integrated passive devices (IPDs) and surface-mounted devices (SMDs) as the passive matching networks, has become a promising fabrication approach for commercial PAs. This solution will reduce the overall module size and cost significantly while not compromising the performance of the PA. Consequently, it has recently received extensive attention from both industry and academia [4,5,6]. The matching networks in [4] and [6] were designed using SMDs, but the presence of the post-matching network resulted in a less concise combiner. The matching network in [4] was designed using IPDs; however, in PA design, SMDs possess two advantages over IPDs. Firstly, SMD inductors have a higher quality factor, leading to the superior performance of the matching network. Secondly, SMD inductors have a higher rated current, making them more appropriate for high-power base station PA design. Therefore, this paper will investigate how to design a high-performance Doherty PA module using SMDs within a limited area.

**Figure 1:**
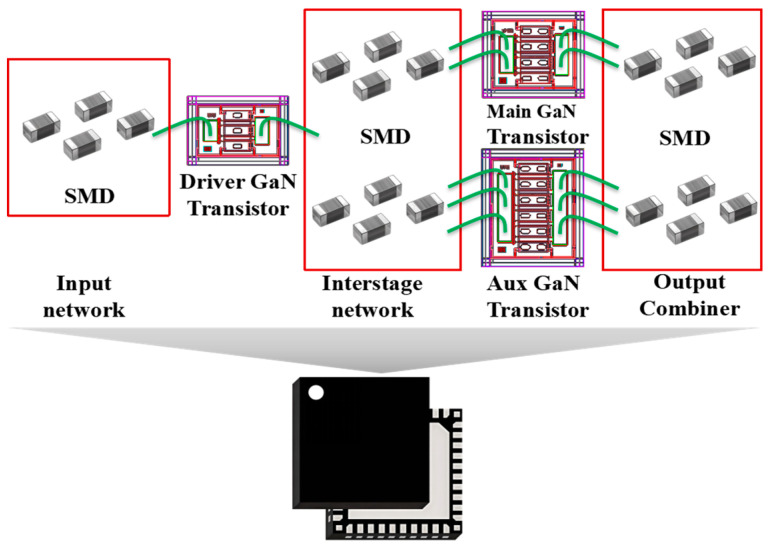
Circuit topology of the two-stage Doherty PA module designed using GaN transistors and SMDs.

This paper presents a method for designing a compact and low-loss combiner suitable for the Doherty PA module. The proposed combiner is applied to a two-stage Doherty PA module designed with GaN transistors and SMDs, as depicted in Figure 1. The GaN transistors and SMDs are assembled on an 8 mm × 8 mm polyimide substrate and integrated into the land grid array (LGA) package. GaN transistors and SMDs are connected through bonding wires, where the two ends of the bonding wires are bonded to the pads on the GaN transistors and substrate, respectively. The proposed DPA module can achieve 29.6–30.4 dB small-signal gain and 40.4–41.1 dBm saturated output power among 3.2–3.6 GHz. The measured saturation drain efficiency (DE) and 8 dB power back-off (PBO) DE reach 51–56.6% and 45.5–48.6%, respectively.

## 2. Circuit Analysis and Design

### 2.1. Analysis and Design of the Output Combiner

**Figure 2:**
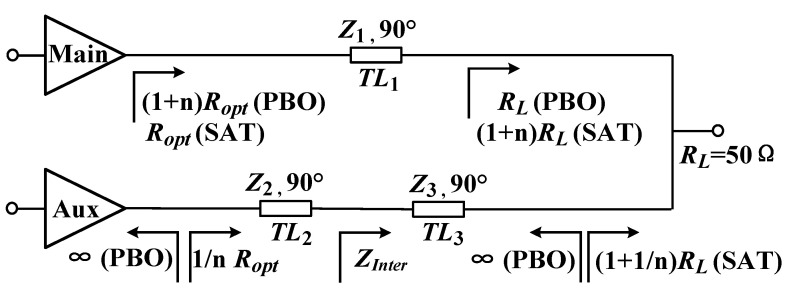
Doherty PA combiner structure based on three quarter-wave transmission lines.

To achieve better efficiency and bandwidth performance, a Doherty power combiner structure based on three quarter-wave transmission lines was selected, as illustrated in Figure 2. Considering that there were two quarter-wave transmission lines following the auxiliary power amplifier in this structure, the impedance at the combination point could be directly set to 50 Ω, regardless of the optimal load impedance *R_opt_* of the main power amplifier. This eliminated the necessity for a post-matching network. The *n* in the figure refers to the ratio of the transistor size between the auxiliary power amplifier and the main power amplifier.

The impedance of each node of the main and auxiliary power amplifiers in the saturation and back-off regions is marked in Figure 2. The characteristic impedance of the quarter-wave transmission line *TL*_1_ could be obtained as follows:

(1) $$Z_{1}=\sqrt{(1+n)R_{L}R_{opt}}$$

To obtain the widest bandwidth, the impedance of the middle node of the two quarter-wavelength lines after the auxiliary power amplifier was selected as follows [7,8]:

(2) $$Z_{Inter}=\sqrt{(1+1/n)R_{L}\times \frac{R_{opt}}{n}}=\frac{\sqrt{(1+n)R_{L}R_{opt}}}{n}$$

Therefore, the characteristic impedance of the quarter-wave transmission lines *TL*_2_ and *TL*_3_ could be obtained as follows:

(3) $$Z_{2}=\sqrt{\frac{R_{opt}Z_{Inter}}{n}}$$

(4) $$Z_{3}=\sqrt{(1+1/n)R_{L}\times Z_{Inter}}=\sqrt{\frac{(1+n)R_{L}Z_{Inter}}{n}}$$

### 2.2. Design Procedure of the Combiner

Considering that a large size of transmission lines is not conducive to implementation inside a PA module of miniaturized size, we hereby propose to use lumped components to achieve the above structure.

**Figure 3:**
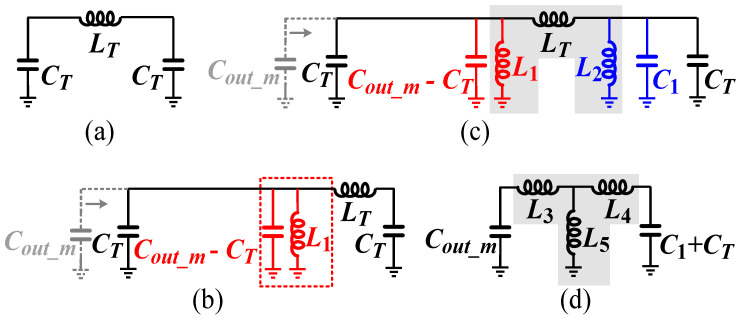
Design procedure of the TL1 implemented with lumped components: (**a**) quarter-wave transmission line modeled by a π-type network; (**b**) π-type network with *Cout_m* absorbed; (**c**) inserting LC resonant network to prepare for the next step; and (**d**) use Δ-Y conversion to obtain a T-type network.

Taking quarter-wave transmission line *TL*_1_ after the main power amplifier as an example, it can be modeled by a π-type network at the center frequency of *f*_0_, as shown in Figure 3a. The component values of the *L_T_* and *C_T_* are calculated as follows [9]:

(5) $$L_{T}=\frac{Z_{1}}{2\pi f_{0}}$$

(6) $$C_{T}=\frac{1}{2\pi f_{0}Z_{1}}$$

The output capacitance of the main PA *C_out_m_* is absorbed into the impedance transformation network to reduce its degradation of bandwidth [10]. According to the load–pull results for the main PA, the *C_out_m_* is larger than the required *C_T_*. Therefore, a short inductor *L*_1_ is needed to resonate out the remaining *C_out_m_* − *C_T_* as shown in Figure 3b. The inductance of the *L*_1_ can be calculated as follows:

(7) $$L_{1}=\frac{1}{{\left(2\pi f_{0}\right)}^{2}(C_{out\_m}-C_{T})}$$

Due to the particularity of the module form, the first matching component of the impedance transformation network must be a series bonding-wire inductor. Therefore, the impedance transformation network must be transformed from a π-type network to a T-type network. To make up for the π-type network, an LC resonant network is inserted into the impedance transformation network as shown in Figure 3c. The capacitance *C*_1_ and the inductance *L*_2_ can be designed to have a parallel resonance at the operating frequency *f*_0_:

(8) $$f_{0}=\frac{1}{2\pi \sqrt{L_{2}C_{1}}}$$

By applying a Δ-Y transformation, the structure of the impedance transformation network is presented in Figure 3d. The corresponding relationships between the inductance values before and after the transformation are as follows:

(9) $$L_{3}=\frac{L_{T}\times L_{1}}{L_{T}+L_{1}+L_{2}}$$

(10) $$L_{4}=\frac{L_{T}\times L_{2}}{L_{T}+L_{1}+L_{2}}$$

(11) $$L_{5}=\frac{L_{1}\times L_{2}}{L_{T}+L_{1}+L_{2}}$$

From Equations (9)–(11), it can be seen that, after transforming from the π-type network to the T-type network, the inductance values of *L*_3_ − *L*_5_ decrease compared to *L*_1_, *L_T_*, and *L*_2_, respectively. For SMDs, smaller inductance values result in a higher quality factor and a higher rated current, making the T-type network more conducive to achieving a high-performance DPA module. The most critical parameter among the above is *L*_3_, which is limited by the design rules of the bonding wire. Therefore, it needs to be designed to a reasonable value first. Next, based on the load–pull results and Equations (1)–(11), the parameters of the entire impedance transformation network can be determined.

**Figure 4:**
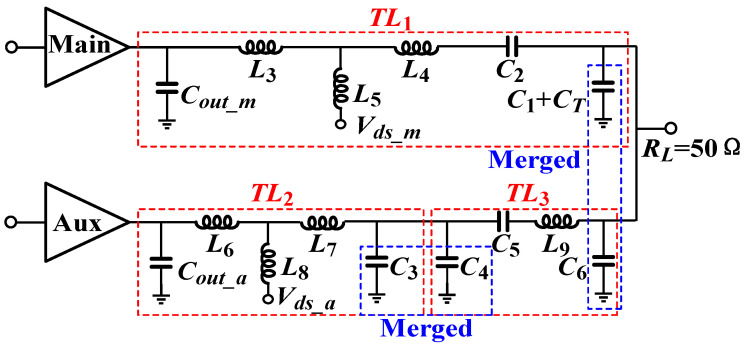
The schematic of the proposed compact and low-loss combiner.

**Figure 5:**
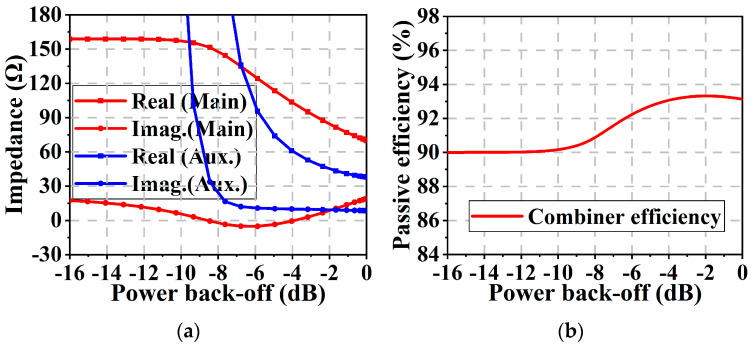
(**a**) Simulated load impedance of the proposed combiner at 3.4 GHz. (**b**) Passive efficiency of the combiner during load modulation at 3.4 GHz.

The analysis method of using lumped components to replace transmission line *TL*_2_ is similar to that of *TL*_1_. The transmission line *TL*_3_ is equivalenced by adopting a π-type network. The final DPA output combiner is depicted in Figure 4. Inductors *L*_5_ and *L*_8_ serve as power supply inductors for the main and auxiliary power amplifiers, respectively. Capacitors *C*_2_ and *C*_5_ serve as DC-block capacitors. The shunt-lumped capacitors at the same node can be merged to simplify the circuit. The simulated load impedances and the passive efficiency of the proposed combiner are plotted in Figure 5. It can be observed that the proposed combiner maintains a passive efficiency of more than 90% while realizing the desired Doherty active-load modulation. In our study, the passive efficiency was simulated as the output power on the load divided by the sum of powers from the main and auxiliary transistor devices delivered into the combiner. All SMDs were simulated using model data provided by the manufacturer with the limited Q value.

### 2.3. Design of Input Matching and Power Divider

**Figure 6:**
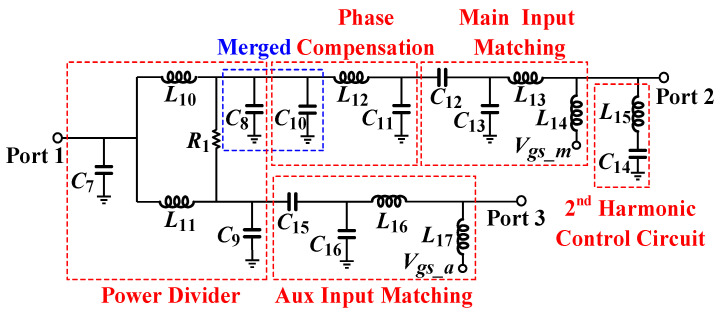
The input matching network and power divider of the DPA module.

The input matching network and the power divider of the DPA module are depicted in Figure 6. The input matching of the main amplifier and the auxiliary amplifier both adopt a multistage band-pass network to expand the bandwidth. A π-type phase compensation network is inserted in the input path of the main PA to align the phase of the main PA and the auxiliary PA at the combination node.

A Wilkinson divider created using lumped components is used for power splitting. The shunt-lumped capacitors at the same node can be merged. A second harmonic control circuit is inserted at the input of the main power amplifier for high efficiency [11]. In addition, a driver stage PA is connected to the input of the Wilkinson power divider for high gain.

## 3. Implementation and Measurement Results

**Figure 7:**
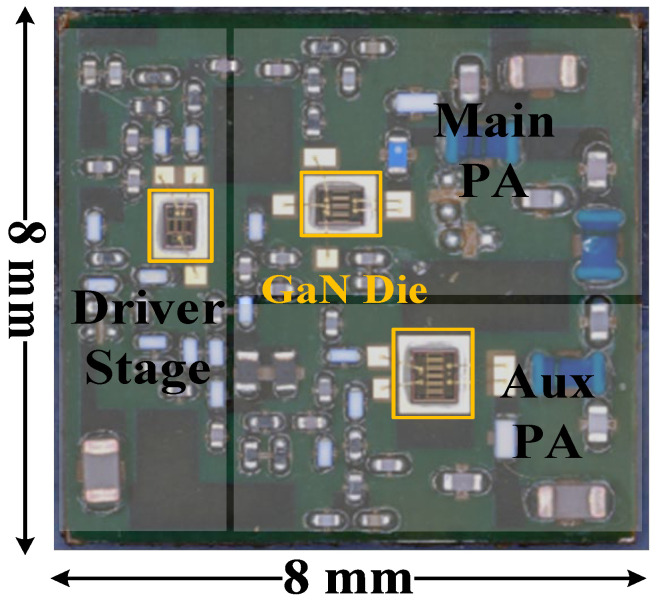
Photograph of the fabricated two-stage DPA module before molding.

The proposed two-stage Doherty PA module was fabricated on polyimide substrate using GaN transistors and SMDs, as shown in Figure 7. The size of this Doherty PA module was only 8 × 8 mm^2^, including the driver amplifier, main PA, and auxiliary PA. The drain bias voltages of the driver, the main PA, and the auxiliary PA were all set to 23 V. The main amplifier and driver amplifier were both biased at Class-AB mode, and the auxiliary amplifier was biased at Class-C mode. The performance of the Doherty PA module was characterized by small-signal, large-signal, and modulated-signal measurements.

### 3.1. Small-Signal Measurement

**Figure 8:**
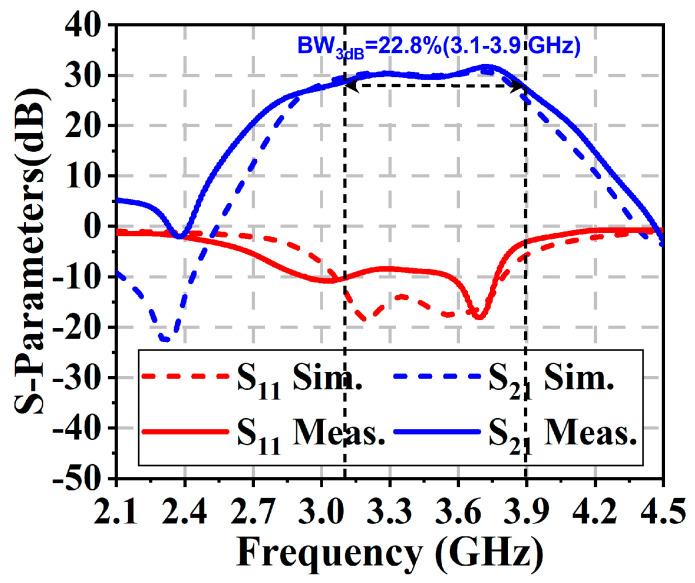
Measured and simulated S-parameters.

Figure 8 compares the simulated S11/S21 parameters with the measured ones. Good agreement can be observed between the simulation and measurement results. The proposed two-stage DPA achieved a 3 dB small-signal bandwidth of 3.1–3.9 GHz and a peak gain of 31.7 dB at 3.71 GHz. The gain fluctuation within 3.2–3.6 GHz was less than 0.8 dB. The measured return loss was about −10 dB within the target band.

### 3.2. Large-Signal Measurement

**Figure 9:**
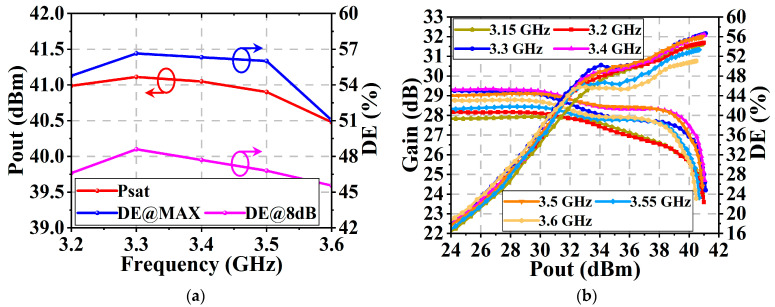
(**a**) Pulsed CW measurements of Psat, peak, and 8 dB back-off DE. (**b**) Measured DE and gain versus output power at different frequencies.

The large-signal performance of the fabricated DPA module was evaluated using pulsed continuous-wave (CW) signal. According to Figure 9a, the proposed two-stage Doherty PA could achieve 40.4–41.1 dBm saturated output power at 3.2–3.6 GHz. The measured saturated DE and 8 dB PBO DE reached 51–56.6% and 45.5–48.6%, respectively. Figure 9b presents the measured drain efficiency and power gain versus output power, respectively. Table 1 summarizes the performance of the proposed DPA module together with state-of-the-art GaN DPA modules/MMICs for 5G base station applications for comparison.

The fabricated two-stage DPA module exhibited excellent comprehensive performance, including the back-off DE, gain, and bandwidth. Compared to state-of-the-art GaN DPA modules/MMICs, the proposed two-stage DPA module exhibited the highest back-off DE, including during the driver stage.

### 3.3. Modulated-Signal Measurement

**Figure 10:**
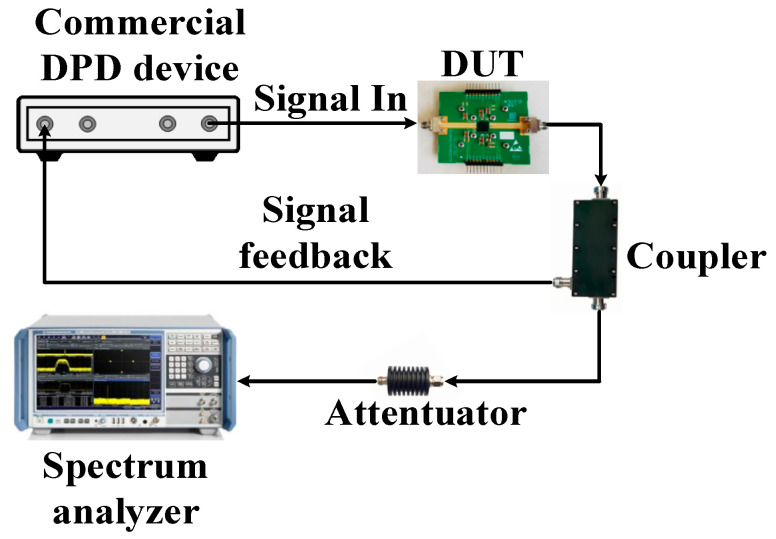
Modulated-signal measurement setup.

**Figure 11:**
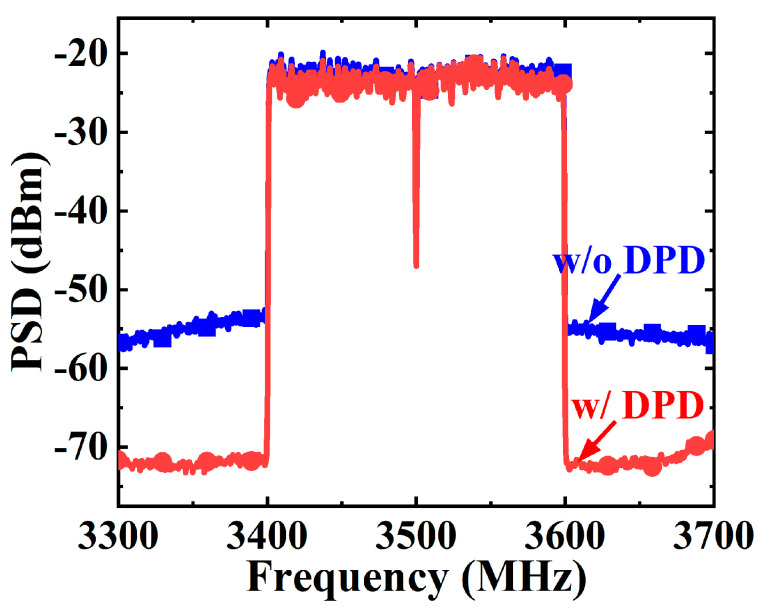
Output PSD with and without DPD at 3.5 GHz.

The experimental setup for the modulated measurements is presented in Figure 10. A commercial digital pre-distortion (DPD) device designed for small-cell base stations was utilized to evaluate the linearity performance of the proposed DPA module. The modulated-signal measurements were carried out using a two-carrier 200 MHz 5G NR signal with a PAPR of 8.8 dB. The DPA module was operated at 10.2 dB back-off to deliver 30.7 dBm average output power and 38.9% drain efficiency at 3.5 GHz. Figure 11 depicts the power spectral density (PSD) of the DPA module’s output signal with and without DPD linearization. Before DPD, the fabricated DPA module achieved good original linearity with a −32.1/−33 dBc adjacent channel leakage ratio (ACLR) performance, which could be improved to −49.2/−48.3 dBc after DPD linearization.

## 4. Conclusions

In this paper, the design procedure and measurements of a high-performance two-stage Doherty PA module are presented, satisfying the requirements of a 5G application. The DPA module is implemented by GaN transistors and SMDs on polyimide substrate with an LGA package size of 8 × 8 mm^2^. The DPA module achieves a small-signal gain of 29.6–30.4 dB and a saturated output power of 40.4–41.1 dBm in the frequency range of 3.2–3.6 GHz. From 3.2 to 3.6 GHz, the designed DPA module provides 51% to 56.6% DE at saturation output power and 45.5% to 48.6% DE at 8 dB of output power back-off. When feeding a two-carrier 200 MHz 5G NR signal, the DPA module achieves a DE of 38.9% at an average output power of 30.7 dBm at 3.5 GHz. The ACLR performance can be improved from −32.1/−33 dBc to −49.2/−48.3 dBc through DPD linearization. Compared to state-of-the-art GaN DPA modules/MMICs, the fabricated two-stage DPA module exhibits excellent comprehensive performance, including peak DE, back-off DE, gain, and bandwidth.

## Figures and Tables

**Table 1:** Comparison with state-of-the-art 3.5 GHz GaN Doherty PAs.

Ref.	Freq.(GHz)	Gain (dB)	Psat (dBm)	DE@OPBO (%)	Stage	Area (mm^2^)	Type
This work	3.2–3.6	29.6–30.4	40.4–41.1	45.5–48.6 @8dB OPBO	2	8 × 8	SMD Module
[3]	3.4–3.8	26.9–28.6	37.7–38.5	42.9–47.8 * @8dB OPBO	2	10 × 6	SMD + IPD Module
[4]	3.4–3.8	10.5–12 **	44.4–45.1	45–49 @8dB OPBO	1	5.6 × 4.3	IPD Module
[5]	3.4–4.1	31.7	47.9	42–44.6 * @8dB OPBO	2	76	Module
[12]	3.3–3.8	12	41.8–42.6	42–51 @6dB OPBO	1	2.4 × 2.4	MMIC
[13]	3.4–3.7	11.7	38.8	40–46 * @8.5dB OPBO	1	10	MMIC
[14]	3.35–3.6	12.1–12.3	43.8–44.7	41–46 @6dB OPBO	1	2.8 × 3.5	MMIC
[15]	3.3–3.6	11 **	42.6–43	34.7–39 @6dB OPBO	1	3.3 × 3.2	MMIC

* PAE. ** Graphically estimated.

## Data Availability

The original contributions presented in the study are included in the article, further inquiries can be directed to the corresponding author.
